# *PIK3CA* Mutational Analysis of Parathyroid Adenomas

**DOI:** 10.1002/jbm4.10360

**Published:** 2020-04-13

**Authors:** Aaliyah Riccardi, Carolina Lemos, Ryan Ramos, Justin Bellizzi, Kourosh Parham, Taylor C. Brown, Reju Korah, Tobias Carling, Jessica Costa‐Guda, Andrew Arnold

**Affiliations:** ^1^ Center for Molecular Oncology University of Connecticut School of Medicine Farmington CT USA; ^2^ Division of Otolaryngology University of Connecticut School of Medicine Farmington CT USA; ^3^ Yale Endocrine Neoplasia Laboratory, Department of Surgery Yale School of Medicine New Haven CT USA; ^4^ Department of Surgery Washington University School of Medicine St. Louis MO USA; ^5^ Center for Regenerative Medicine and Skeletal Development, Department of Reconstructive Sciences University of Connecticut School of Dental Medicine Farmington CT USA; ^6^ Division of Endocrinology and Metabolism University of Connecticut School of Medicine Farmington CT USA

**Keywords:** PARATHYROID‐RELATED DISORDERS, DISORDERS OF CALCIUM/PHOSPHATE METABOLISM, CANCER

## Abstract

Benign parathyroid adenoma is the most common cause of primary hyperparathyroidism, whereas malignant parathyroid carcinoma is exceedingly rare. Distinguishing parathyroid carcinoma from benign adenoma is often difficult, and may be considerably delayed even after surgical resection until the rigorous diagnostic criteria of local invasion of surrounding tissues and/or distant metastases are fulfilled. Thus, new insights into their respective molecular bases may potentially aid in earlier diagnostic discrimination between the two, as well as informing new directions for treatment. In two recent studies, gain‐of‐function mutations in *PIK3CA*, a recognized driver oncogene in many human malignancies, have been newly identified in parathyroid carcinoma. To assess the potential specificity for malignant, as opposed to benign parathyroid disease, of *PIK3CA* hotspot mutations, we PCR‐amplified and Sanger sequenced codons 111, 542/545, and 1047 and the immediate flanking regions in genomic DNA from 391 typical, sporadic parathyroid adenomas. Four parathyroid adenomas (1%) had subclonal, somatic, heterozygous, activating *PIK3CA* mutations. The rarity of *PIK3CA* activating mutations in benign parathyroid adenomas suggests that tumorigenic activation of *PIK3CA* is strongly associated with malignant parathyroid neoplasia. However, it does not appear that such mutations, at least in isolation, can be relied upon for definitive molecular diagnosis of parathyroid carcinoma. © 2020 The Authors. *JBMR Plus* published by Wiley Periodicals, Inc. on behalf of American Society for Bone and Mineral Research.

## Introduction

Benign parathyroid adenoma is responsible for the large majority of primary hyperparathyroidism (PHPT) cases, whereas parathyroid carcinoma is rare, accounting for <1% of PHPT. Because of histologic similarities, it can be difficult to distinguish parathyroid carcinoma from its malignant counterpart, and a diagnosis of carcinoma can only be made when there is clear evidence of invasive growth into adjacent structures and/or distant metastasis.^1^ The *MEN1* tumor suppressor gene and the *CCND1* oncogene (encoding cyclin D1) are well‐established drivers of parathyroid adenoma^2^ and lower frequency mutations have been described in other candidate driver genes, such as *EZH2*,^3^ *ZFX*,^4^ and several cyclin‐dependent kinase inhibitors in parathyroid adenomas.^2^ The *CDC73/HRPT2* tumor suppressor gene is the most frequently altered driver gene in parathyroid carcinoma^2^; amplifications of *CCND1* have also been observed.^5^, ^6^ Furthering the frontier of knowledge regarding the genetic changes that contribute to parathyroid adenoma or carcinoma, separately, could allow for early distinction between the two and lead to treatments that target these variants specifically.

**Table 1:** Primers Used for PCR and Sequencing

Mutational hotspot	Forward primer (5′ to 3′)	Reverse primer (5′ to 3′)	Expected product size (bp)
K111E	CCTCCATCAACTTCTTCAAG	ATAGTTCCATAGTTCGATAG	346
E542K/E545K	GTAAATCATCTGTGAATCCAG	CTGCTTTATTTATTCCAATAG	310
H1047R	GGTTTCAGGAGATGTGTTAC	TGGATTGTGCAATTCCTATGC	372

**Table 2:** Clinical and Histopathologic Details for PIK3CA‐Mutation Positive Cases

Tumor	Mutation	Estimated allelic fraction	Weight (mg)	Size (greatest dimension, cm)	Oxyphil cell content	Preoperative serum calcium (mg/dL)	Preoperative serum PTH (pg/mL)
1	E542K	0.219	475	1.5	25%	10.2	97
2	E542K	0.154	998	1.8	70%	10.9	131
3	H1047R	0.200	380	1	<1%	11.3	110
4	H1047R	0.286	137	0.6	5%	Not available	106

**Figure 1:**
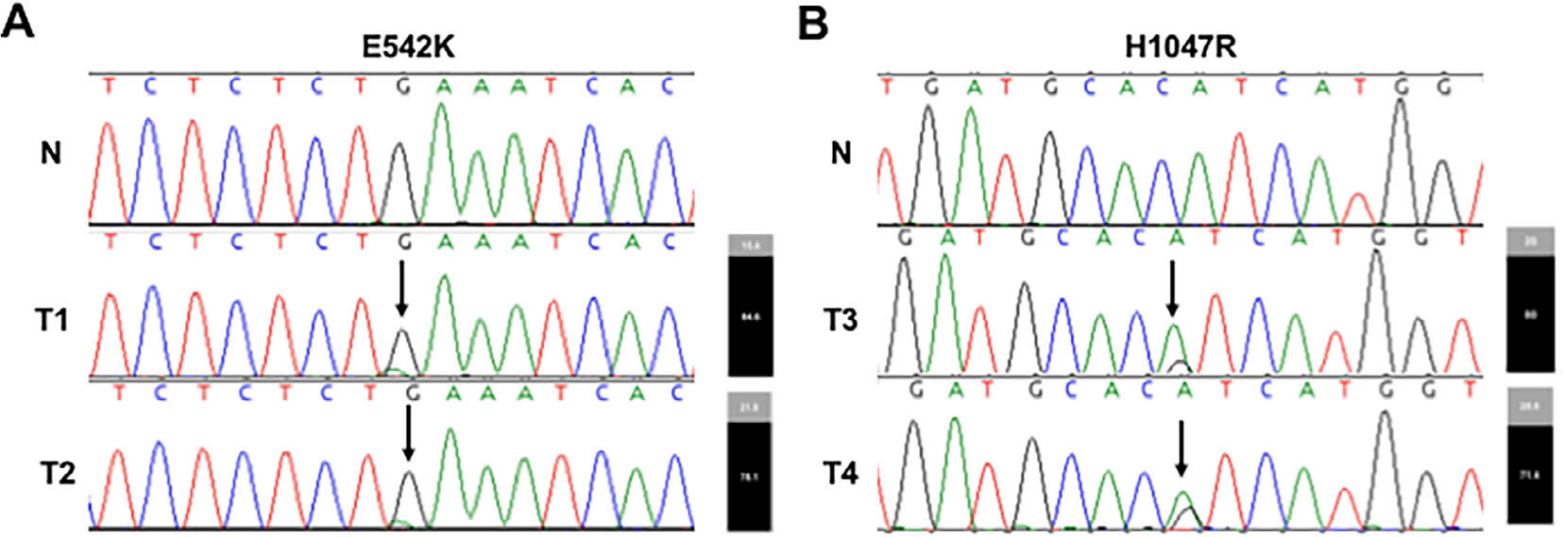
Subclonal, activating *PIK3CA* hotspot mutations in sporadic parathyroid adenomas. (*A*) E542K mutation. In the normal sequence (N) from a wild‐type (WT) control DNA sample, a single guanine (consistent with homozygosity) is present. The presence of an additional, mutant adenine base as a heterozygous, subclonal change in two tumor samples (T1 and T2) is indicated by an arrow. The proportion of mutant alleles (gray) relative to WT alleles (black) obtained by sequencing of subcloned PCR products is shown in the bar graphs to the right of the chromatograms. (*B*) H1047R mutation. In the normal sequence (N) from a WT control DNA sample, a single adenine is present. The presence of an additional, mutant guanine base as a heterozygous, subclonal change in two tumor samples (T3 and T4) is indicated by an arrow. The proportion of mutant alleles (gray) relative to WT alleles (black) obtained by sequencing of subcloned PCR products is shown in the bar graphs to the right of the chromatograms.

*PIK3CA* encodes the p110 alpha catalytic subunit of phosphatidylinositol 3‐kinase (PI3K), a member of the *PI3K/MTOR/AKT* pathway, which has important roles in regulating cell growth and survival, and cell cycle progression, frequently subject to alteration in human tumors. Once activated, PI3K converts PIP2 to PIP3 via phosphorylation. PIP3 activates AKT via phosphorylation, which then activates mTOR leading to cellular growth, increased survival, and angiogenesis.^7^ Recent studies have reported *PIK3CA* mutations in approximately 10% of parathyroid carcinomas.^6^, ^8^, ^9^, ^10^ These mutations, p.K111E, p.E545K, and p.H1047R, are identical to activating, hotspot mutations known to be involved in other human cancers, including breast, lung, ovarian, and colorectal cancers.^11^ Activating mutations of *MTOR*, also commonly altered in human tumors, were also seen in parathyroid carcinomas in these studies, more broadly suggesting the likely importance of alterations of the PI3K/MTOR/AKT pathway in malignant parathyroid tumorigenesis.^6^, ^8^

With this mounting evidence for the contribution of *PIK3CA* activating variants to parathyroid neoplasia, we set out to assess if such mutations are present in parathyroid adenomas and to thus evaluate their potential specificity for malignant parathyroid disease.

## Materials and Methods

### Patients and samples

We analyzed 391 tumor samples from patients undergoing parathyroidectomy for primary hyperparathyroidism. All tumors included in this study were typically presenting, sporadic adenomas, meaning disease was restricted to a single gland with no evidence of recurrence, and no family history or personal history of hyperparathyroidism. Histologic examination revealed no malignant and/or atypical features and a high degree of tumor purity. All samples were obtained with informed consent in concordance with IRB‐approved protocols. The tumor genomic DNA was extracted from flash frozen tissue using proteinase K digestion and subsequently a phenol–chloroform extraction and ethanol precipitation. When available, matched nontumor control DNA was obtained from blood or other nontumor tissue samples from the corresponding patients.

### Polymerase chain reaction and Sanger sequencing

We designed primers to cover the three mutational hotspots previously implicated in parathyroid tumorigenesis: p.K111E, p.E545K, and p.H1047R (Table 1); primers for the p.E545K mutation were designed to also cover the p.E542K mutation, involved in other cancers. PCR was performed in 20‐μL reaction volumes, each containing 25 ng of extracted tumor DNA, 12 μL of H_2_O, 2 μL 10X PCR buffer, 200μM of deoxynucleotide triphosphates, 1.5μM of MgCl_2_, 1μM of each primer, and 1 unit of AmpliTaq Gold (Applied Biosystems/ThermoFisher, Waltham, MA, USA). The reactions were performed by incubating at 95°C for 10 minutes, followed by 35 cycles of 95°C for 30 s, 55°C for 30 s, and 72°C for 1 min, and a final elongation step of 72°C for 10 min. PCR products were purified with ExoSAP‐IT (Affymetrix, Santa Clara, CA, USA) and sequenced by GENEWIZ (GENEWIZ, Inc, South Plainfield, NJ, USA). The resulting sequencing data were analyzed and compared with the published reference sequence (ENST00000263967.3). An independent PCR and sequencing reaction confirmed any variants. For tumor samples containing a *PIK3CA* mutation, corresponding nontumor germline DNA (when available) was also sequenced to determine the germline/somatic status of the mutation. Selected PCR products were subcloned using the TOPO TA Cloning Kit for Sequencing (Invitrogen, Waltham, MA, USA). DNA was extracted from 10 to 20 colonies using the QIAprep Spin MiniPrep Kit (Qiagen, Germantown, MD, USA) and processed for sequencing by GENEWIZ.

## Results

We analyzed a series of 391 parathyroid neoplasia samples for mutations in *PIK3CA* mutational hotspots and identified mutations in four typically presenting sporadic parathyroid adenomas (Fig. 1). p.E542K was identified in two tumor samples; germline DNA was not available from either sample, so the germline/somatic status of the mutation could not be confirmed. p.H1047R was identified in two additional tumor samples, and in both cases, was absent from the patients' germline DNA, confirming that p.H1047R was somatic. Interestingly, in all four tumors, the mutant allele peak was smaller than the retained wild‐type (WT) peak. Subcloning of the PCR products revealed that the p.E542K mutation was present in 15.4% (4/26) and 21.9% (7/32) of sequenced clones; the p.H1047R mutation was present in 20% (4/20) and 28.6% (6/21) of sequenced clones. Clinical and histopathologic details for *PIK3CA*‐mutation positive cases are provided in Table 2.

## Discussion

Recent evidence has suggested that alterations of the PI3K/MTOR/AKT pathway, and particularly activating, hotspot mutations of *PIK3CA*, may play an important role in malignant parathyroid tumorigenesis. In the present study, we explored the role of specific *PIK3CA* gain‐of‐function mutations in a large series of typical, benign sporadic parathyroid adenomas to assess the potential specificity of such mutations for malignant, as opposed to benign, parathyroid tumors. We identified activating *PIK3CA* hotspot mutations in four parathyroid adenomas (1%). These mutations were confirmed to be somatic in both cases where germline control DNA was available, and the remaining two are almost certainly somatic as well because germline activating *PIK3CA* hotspot mutations have never been described in tumors.^12^

The most intriguing feature of the identified *PIK3CA* mutations is that, in all four parathyroid adenomas, observed *PIK3CA* mutations were subclonal and likely present in only a subpopulation of tumor cells. First, in sequencing chromatograms, the relative heights of the mutant peaks were much lower than that of the WT peak at that position. In contrast, despite the semiquantitative nature of Sanger methodology, in our experience sequencing parathyroid adenomas the observed peak height ratio for heterozygous variants is typically very close to 1:1,^4^, ^13^ reflecting the exceedingly high tumor cell purity characteristic of parathyroid adenomas. Next, the proportion of mutation‐containing clones (15.4% to 28.6%) obtained by sequencing of subcloned PCR products was consistent with the relative peak heights observed in Sanger sequencing of the original tumor sample. Further, all four mutation positive cases were re‐examined by experienced endocrine pathologists, confirming a high degree of tumor purity, therefore ruling out the possibility that subclonal mutations was caused by normal cell contamination. The presence of *PIK3CA* mutations at allelic fractions of less than 50% suggests that *PIK3CA* mutations are not present in all or most tumor cells. It is possible that the presence of an activating *PIK3CA* mutation may confer malignant potential on a parathyroid cell and that the subpopulations of cells harboring *PIK3CA* mutations may have been capable, if given enough time and additional genetic alterations, of progressing to parathyroid cancer. Data suggest, however, that except in certain circumstances such as germline *CDC73* mutation, parathyroid carcinoma likely arises de novo, rather than through a benign adenoma intermediate.^14^ Subclonal *PIK3CA* mutations may therefore be insufficient to drive malignant transformation in the context of an established benign parathyroid adenoma cell, perhaps requiring another, rare cooperating gene mutation to confer invasive potential. Although established, oncogenic *PIK3CA* hotspot mutations are significantly more common in parathyroid carcinoma (approximately 10%) in comparison to parathyroid adenomas (1%; Fisher exact test, *p* = 0.0019), adenomas are roughly 100 to 200 times more common than carcinoma, decreasing the positive predictive value of *PIK3CA* mutational testing in unselected cases. This positive predictive value would be even lower if our methodology failed to detect some additional adenomas containing tiny *PIK3CA* mutant subclones below the method's threshold of sensitivity. In the absence of any other criteria suggestive of malignancy, and we emphasize that the four mutation‐positive benign adenomas in this study had no atypical clinical and/or histopathologic features, an activating *PIK3CA* hotspot mutation alone does not appear to be a robust predictor of parathyroid malignancy.

In the context of parathyroid cancer, the prevalence of activating PI3K/AKT/mTOR pathway mutations in a substantial subset of patients provides a potential “actionable target” for therapeutic intervention, given that a number of PI3K pathway inhibitors have already been developed. Thus, tumor tissue from patients with surgically incurable parathyroid cancer can rationally be subjected to DNA sequencing, with patients proving to harbor clonal somatic activating *PIK3CA* mutations being considered for appropriately targeted treatment,^2^, ^6^ ideally in the setting of a study. Such an approach also applies to other putatively actionable targets,^2^, ^6^ and ultimately clinical trials will be needed to validate efficacy. Further studies, particularly those demonstrating the ability of activating *PIK3CA* mutations to experimentally drive primary hyperparathyroidism, would be valuable for providing a model system in which the basis of malignant behavior in the parathyroid cell context can be investigated.

## Disclosures

The authors have no conflicts of interest to declare.
